# Birthing #blackboyjoy: Black Midwives Caring for Black Mothers of Black Boys During Pregnancy and Childbirth

**DOI:** 10.1007/s10995-021-03224-1

**Published:** 2021-08-27

**Authors:** Keisha L. Goode, Arielle Bernardin

**Affiliations:** 1 State University of New York College at Old Westbury, Old Westbury, NY USA; 2 National Association of Certified Professional Midwives, Keene, NH USA; 3 The Birth Place Lab, University of British Columbia, Vancouver, BC Canada; 4 New York, NY USA; 5 Freeport, NY USA

**Keywords:** Black, Midwifery, Motherhood, Boyhood, Reproductive justice

## Abstract

**Background:**

Structural racism mediates all aspects of Black life. The medicalization of pregnancy and childbirth, and its detrimental impacts on Black birth, is well documented. The Black Lives Matter movement has elevated the national consciousness on all aspects of Black life, but significant attention has been directed toward the murder and dehumanization of Black men and boys. Black midwives, caring for Black people, using the Midwives Model of Care© which consistently demonstrates its efficacy and better outcomes for Black people, are uniquely positioned to witness the physical and psychosocial experiences of birthing Black boys in America.

**Methods:**

Between 2011 and 2013, the first author conducted interviews with 22 Black midwives to understand their perceptions of, and experiences in, predominantly white midwifery education programs and professional organizations. Convenience and snowball sampling were used. This paper investigates previously unreported and unexamined data from the original study by focusing on the witness and insight of nine midwives who provided care for Black mothers of boys during pregnancy and childbirth.

**Findings:**

The data presented three themes: It’s a Boy: On Restlessness and Complicated Uneasiness; Desensitization of Black Death; and, Physiological Impacts of Toxic Stress.

**Conclusions:**

The findings demonstrate that caring for Black people must be simultaneously theorized and executed within an anti-racist, relationship-centered, reproductive justice framework. Black midwives are uniquely positioned to do this work. Greater attention, in practice and in research, is needed to investigate the birth experiences of Black mothers of boys.

## Significance

The Black birth crisis, and the crisis of structural racism and violence leading to the dehumanization and murder of Black men and boys, are simultaneous and intersecting. This study investigates the experiences of Black midwives caring for Black mothers of Black boys throughout pregnancy and childbirth. The physical and psychosocial impacts of structural racism are explored to frame the necessity of: increasing the number of Black midwives in the United States; researching the experience of birthing Black boys in America; and, caring for Black people in an anti-racist, relationship-centered, reproductive justice framework.

## Introduction

Black motherhood is mediated by social institutions that foster or disallow life. Too often, Black motherhood is embedded with a fear that they, or their infants, may not survive pregnancy and childbirth because the dominant U.S. medical model prioritizes capitalist values of industrialization and systemization over the physical and psychosocial well-being of Black birthing people and infants. Further, Black men are approximately two and half times more likely to be killed by police in their lifetime than are white men (Edwards et al., 2019). Black motherhood’s fear is additive and multiplicative. Birthed as a hashtag on social media in 2016, #blackboyjoy is illustrated in images of Black boys and men smiling, laughing, creating, and at play. Black people exalt and celebrate #blackboyjoy in communities, on social media and in popular culture to reclaim and honor the fullness and humanity of Blackness.

The Midwives Model of Care©, inherently relationship-centered, consistently proves to be safer, more cost effective, and more attentive to the physical and psychosocial needs of birthing people (Vedam et al., 2018; Yoder & Hardy, 2018).The model centers on “monitoring the physical, psychological, and social well-being of the mother throughout the childbearing cycle; providing the mother with individualized education, counseling, and prenatal care; continuous hands-on assistance during labor and delivery, and postpartum support; minimizing technological interventions; and, identifying and referring women who require obstetrical attention.”^1^ The values of the medical model of pregnancy, reflective of U.S. capitalist and white supremacist culture, are counter to the process of physiologic birth. This model leads to unnecessary interventions that are dangerous for all, but especially Black birthing people (Wolf, 2018). As Petersen et al. (2019) note, Black people are two to three times more likely to die from pregnancy-related causes than white people. Black people with at least a college degree are still five times more likely to die than their white counterparts (Petersen et al., 2019).

Among people over age 30, Black people’s likelihood of pregnancy-related death increases to four to five times higher than white people.

In a society built upon structural racism, race matters. Concordant care matters (Janevic et al., 2020). This study, using unexamined qualitative data from the first author’s dissertation, centers the experience of nine U.S. Black midwives bearing witness to Black mothers of boys during pregnancy and childbirth. The following research question was used to guide this study: How do Black midwives witness, interpret, and care for the physical and psychosocial impacts of toxic stress on Black mothers pregnant with Black boys? We argue that Black midwives are uniquely positioned to support people in birthing #blackboyjoy.

### Theoretical Frameworks

This paper is situated within the theoretical frameworks of Symbolic Interactionism, Critical Race Theory, and Reproductive Justice. Herbert Blumer’s (1986) *Symbolic Interactionism* theorizes that humans act on the basis of meaning derived from social interaction. That is, when one interacts with “X”, they are also interacting with the meaning attached to “X.” Those meanings are in constant flux, as meanings are an emergent property.

Critical Race Theory examines white supremacy and macro and micro manifestations of systemic racism (Crenshaw et al., 1995). The tradition places value on experiential knowledge. Black midwives’ experiential knowledge and their meaning-making of the Black body, and Black birth, are central to this study. Such meanings are rooted in understandings of history, racialized trauma and its contemporary manifestations on the Black body.

Reproductive Justice is “the human right to maintain personal bodily autonomy, have children, not have children, and parent the children we have in safe and sustainable communities” (Ross & Solinger, 2017). The care that Black people experience during pregnancy and childbirth is a matter of life or death, as the mortality and morbidity data evidences (Petersen et al., 2019). Further, as the data in this paper supports, Black mothers’ fear of not being able to parent their children in safe and sustainable communities is tangible. We assert that caring for Black pregnant and birthing people must be simultaneously theorized and executed within an anti-racist, relationship-centered, reproductive justice framework.

### Literature Review

Structural racism produces toxic stress. Toxic stress—like economic, food and housing insecurity—is a structural determinant of health. Structural inequities determine, and exacerbate, health disparities (American Psychological Association, 2017).

Brian McEwen’s (1998) work on allostasis, allostatic load and the weathering effect contextualizes the interrelationship between structural racism and toxic stress. Allostasis is the body’s attempts to maintain stability during and after exposure to stressful experiences. The body depends on these systems for regulation and protection from stress. If the body is continuously exposed to prolonged periods of allostasis, it reaches a limit or allostatic load. The load represents the body’s inability to recover from prolonged wear and tear on the system. The load has a “weathering effect” and gradually increases the risk of chronic disease and declining reproductive health. People who report continuous experiences of discrimination exhibit increased stress markers such as slower blood pressure recovery, inflammation, and sleep deprivation (Colen et al., 2019). Further, Black people are disproportionately affected by Perinatal Mood and Anxiety Disorder (PMAD), of which sleep deprivation is a key characteristic (Mitchell et al., 2010).

Violence exposure is a significant toxic stressor. Mothers do not need to directly witness an act of violence to experience “anticipatory hypervigilance” in the face of structural racism and violence (Nuru-Jeter et al., 2009). Johnson et al. (2009) report that people who experience elevated amounts of neighborhood/community violence are more likely to report less than seven hours of sleep per night, as well as interrupted sleep. Further, exposure to violence during gestation is correlated with lower than average birthweight (Bell et al. 2012). Alang et al. (2017) report that being pregnant during the time of one police murder of a Black person accounts for as much as a third of the Black-white gap in birth weight. This violence has intergenerational impacts because studies indicate that early, continuous exposure to stress due to historical and/or personal racialized trauma contributes to increased stress reactivity and subsequent poor physical and mental health outcomes, especially among Black boys (Geronimus et al., 2015; Heard-Garris et al. 2018; Johnson et al., 2013; Staggers-Hakim, 2016).

## Methods

Between 2011 and 2013, as a doctoral candidate in Sociology, the first author conducted one-on-one, semi-structured, audio-recorded interviews with 22 Black midwives. The purpose of the study was to better understand how a racist, sexist and classist denigration of Black midwives in the early twentieth century is still manifesting itself in their experiences in predominantly white midwifery education programs and professional organizations. With just two–three percent of the U.S. midwifery workforce identifying as Black, midwifery is a microcosm of systems that fails to recruit, retain, and support the leadership of Black people. Such failures are structural barriers toward more Black midwives serving more Black birthing people.

The researcher was certified in human subject research through the Collaborative Institutional Training Initiative and studied qualitative research methodology as part of doctoral training and coursework. This study was approved by the first author’s doctoral-granting institution’s Institutional Review Board. Initially, a convenience sample approach was used. Recruitment flyers detailing the study’s research questions were disseminated at a 2010 national midwifery conference and via a national midwifery professional organization email list for midwives of color. This approach yielded just four participant interviews.

In 2010, the researcher had no prior relationship with the Black midwifery community. Thus, trust needed to be earned. The researcher-a Black woman-had done extensive reading on the history of Black midwifery and expressed a commitment to the community beyond doctoral studies. Established trust with the initial four participants led to a snowball sampling approach to identify the remaining study participants. All interview requests were granted. All participants signed an Informed Consent form. 18 of the 22 interviews were in-person. The other four were conducted via phone or Skype. The interviews were, on average, 60–90 min in length. The researcher took field notes throughout the study’s duration. Repeat interviews were not conducted, though the researcher maintained a relationship with participants throughout the study. Interview notes were shared with participants and informed the study’s findings.

Black midwives witnessing the pregnancy and birth of Black mothers was not initially a focus of the research as designed and was not reported in the dissertation. However, Trayvon Martin’s tragic death in 2012 occurred during the interview research. His death reignited national attention to police violence, and the country’s failure to consistently demonstrate that Black lives matter. Since then, many more Black lives have been lost, many of whose names did not make national headlines. The importance of these data and the timeliness of their examination confirms the argument of Schiellerup (2008), who states: “The consideration of data analysis joins the positionality debate in that the possibility of researchers and the social context in which they are undertaking research will influence what strikes them in the data, their decisions about what to include and what to omit, the kinds of stories to tell and not to tell” (p. 163). So, in 2020, at the height of protests in the aftermath of George Floyd’s tragic death, all transcripts and notes with the 22 Black midwives interviewed between 2011 and 2013 were reviewed with this paper’s research question in mind. “Black boy” or “Black son” were specifically referenced in nine of the interviews. The first author preferred to manually engage with the data so qualitative analysis software was not utilized. The first author identified topics and themes in those interviews and then developed codes to reflect the three major themes presented in the Results section of this paper.

Collectively, seven African-American and three Caribbean-American midwives, ranging from five to 40 years of experience, are represented in this sample. Five are Certified Professional Midwives (CPMs) and four are Certified Nurse Midwives (CNMs). CPMs practice in homes, freestanding birth centers or clinics. They may be trained via a competency-based apprenticeship with a seasoned wife or attend an accredited midwifery training program or school. CPMs have a path to licensure in 34 states and the District of Columbia. CNMs primarily practice in hospitals or clinics, though they may also practice in homes and freestanding birth centers. CNMs are Registered Nurses trained in graduate-level midwifery programs. CNMs are legally recognized to practice in every state in the U.S. and in the District of Columbia. To earn either the CPM or CNM credential, successful completion of a certification examination is required, along with recertification every three or five years, respectively.

## Results

The data illustrated three themes: It’s a Boy: On Restlessness and Complicated Uneasiness; Desensitization of Black Death; and, Physiological Impacts of Toxic Stress. Throughout, Black midwives conveyed a sense of understanding, hope and comfort that ground their care in service of a reclamation of joy.

### It’s a Boy: On Restlessness and Complicated Uneasiness

Systems of white supremacy and patriarchy interact to construct the social meanings attached to Black people, especially men and boys. Midwives, given the relationship-centered nature of their care, sensed a certain “restlessness” or “complicated uneasiness” in the mothers of boys they were caring for. Gwendolyn (CNM, 20–25 years of experience) says:

I had a Black mama last week that...wow….just a restlessness was there….you know, just not being able to settle. A first-time mama and I have been with her from the beginning and when, you know, she found out it’s a baby boy…...yeah, there was a shift. It’s like she is scared...beyond the first-time mama stuff. Black babies don’t get to be babies or kids….so I had her imagine laughing and playing with her boy.

Hattie (CPM, 35–40 years of experience) speaks to a Black mother’s “complicated uneasiness” by saying:

Damn. You know, she has a 2-year old son now...he’s tall for his age... and I was her midwife then, too, and that was her first baby….and just the worry that is embedded on her as the mom of Black boys….two….it’s like a complicated uneasiness. She is so happy but there is just an extra worry when it’s a boy...just sometimes teachers, police, even in the store. America is violent. I know as a mom and as a midwife. This is...back to your question….why you need Black midwives…..She comes in and is here for hours. She knows I get her. I love her.

Dorothy (CNM, 5–10 years of experience) wrestles with attempting to mitigate the restlessness and complicated uneasiness that Gwendolyn and Hattie reported. Dorothy asks, “Is it wrong that I often consider telling my Black mamas to just avoid finding out about the sex all together? I wonder…because I can love on them throughout the pregnancy and at the birth…is it good care just to tell them to wait so they just…settle more?….Laugh and be silly with me.”

The questions are justified. Studies demonstrate that Black children are often perceived to be older, and less innocent, than their white peers, further contributing to their dehumanization (Goff et al., 2014). Bell (2017) has theorized the social operation of Black boys as “symbolic assailants,” or how police and other people in power view Black boys as threatening, independent of their behavior. The dehumanization of Black bodies is a historical continuity, to which this study’s participants bore witness.

### Desensitization of Black Death

America’s desensitization to and sensationalism of Black death has been well-documented (Chaney & Robertson, 2015). Midwives illustrate within the context of birth. Kimberlee (CNM, 20–25 years of experience) says:

I have been a midwife for a long time now….I think what I am seeing with my Black moms is just how these deaths of our men aren’t….valued….and we all feel it….they are seeing in to that. You know, it’s….desensitization. It’s just regular. It’s normal. And I think these mamas are sensing in to that whether they say it or not.

Echoring Kimberlee’s thoughts, Octavia (CPM, 15–20 years of experience) explains: “Then there’s America’s obsession with…these gross images of Black death and mostly our Black men and boys. I mean, I think that plays into it to because….it’s no consequence and it’s like America’s porn. Imagine how they feels?”. Zora (CPM,10–15 years of experience) adds: “And you know what…I am a mama of boys myself so when I have a Black mama of a boy…it’s like….I see you, sis. I get you.”

These data illustrate not only the desensitization and sensationalism of Black death in America, but also the voyeuristic nature of the circulation of photographs and videos. On one hand, they have raised national attention to white supremacy and anti-Blackness in America. On the other hand, the circulation of these videos may re-traumatize Black people and allows others to be voyeurs into Black pain. America’s history of lynching Black people is such an illustration, wherein Black slaughter was memorialized in postcards, photographs, and audio-recordings (Waldrep, 2006). These acts suggest a much more active desensitization—Black death becomes sport. The laughter, play and silliness that Black midwives foster, much like the #blackboyjoy imagery, is a celebration of Black life, which is a stark contrast to the desensitization of which they speak.

### Physiological Impacts of Toxic Stress

Midwives’ meaning-making support the literature on the physiological impacts of structural racism and toxic stress.

Audre (CPM, 10–15 years of experience) addresses sleep deprivation throughout pregnancy:

We’re midwives so...you know….we just have a closer relationship with the families because that is our model. And...I get calls and texts like I ‘I can’t sleep’ all the time and I am telling you...that happens more when she is having a boy….you know, Black women. And no sleep is just so bad for the body...especially when pregnant. It’s not that the worry isn’t there for our girls….it’s there but it’s just more...like intense. There is nothing wrong with their body.

bell (CNM, 5–10 years of experience) addresses sleep deprivation, but also anxiety, depression, and high blood pressure throughout pregnancy and the moment of birth:

I have been thinking about this a lot….there’s a pattern there. Same issues: not sleeping, heart being faster than normal, blood pressure up and not going down, just….so damn anxious. And sad. This country does not do right by Black women and it impacts them when they have these boys. And it’s not just the pregnancy...it’s the birth. I don’t know how to explain it even though I have been thinking….you know, about it. It’s like...I give her extra love, encouragement and joy so she knows she can do it and he will be okay. There’s...there’s just a moment.

bell’s words support the literature on PMAD, but “the moment” of birthing Black boys of which she speaks complicates current understanding of the issue.

bell and Patricia (CPM, 20–25 years of experience) are in conversation with each other, although practicing in different settings and on opposite sides of the country. Patricia says:

There’s a lot of stuff out there about Black women and low birthweight and some of it blames the mom. No...it’s the system….it’s racism. You know that very well. And the no sleep, the stress, the anxiety, the high blood pressure….all because of everyday experiences of being Black in America and their baby boys gestate in that. The fear is there throughout the pregnancy…and….I even see it at the birth though I can’t explain it….and even beyond that. I’m a midwife...I get to know these mamas well…and see them afterwards….and the fear is just there and it’s just...so sad. And toxic. But, hey? I do everything to bring a smile...bring joy. And their babies are just fine. The joy matters.

The relationship-centered Midwives Model of Care©, as the data suggests, may serve as a powerful support and intervention to the physiological impacts of toxic stress. Black midwives caring for Black people may be a *protective* determinant of health.

## Discussion

Dumas and Nelson (2016) argue that “Black boyhood is socially unimagined and unimaginable, largely due to the devalued position and limited consideration of Black girls and boys within the broad social conception of childhood….to assert that Black boyhood is unimagined and unimaginable is to lament that we have created a world in which Black boys cannot be” (p. 28). The data herein confirms a persistent overarching fear of both the future that awaits Black boys, as well as a dissociation of Black boyhood with play. Therefore, #blackboyjoy serves as a reminder of such a state of being and a reclamation of the joy of Black boyhood. *Being* is the precursor to the mattering that Black Lives Matters exalts.

The un-imagination is a *socia*l un-imagination, and represents a continuation of the historical dehumanization and de-mattering of Black life (Berry, 2017; Owens, 2017). As Ta-Nehisi Coates (2015) writes, “In America, it is traditional to destroy the black body—it is heritage” (pp. 103–104). Despite the restlessness, uneasiness, and physiological impacts of toxic stress that comes with the desensitization of Black death, Black mothers continue to imagine. They imagine their boys laughing and playing, full of the creativity, discovery, wonderment, and joy that children possess, and for which mothers hope. When we do not foster Black parents’ imaginations, when Black parenthood is intertwined with grief, we cripple Black futures.

Thus, structural racism is a public health issue (Alang et al., 2017; Owens & Fett, 2019). Birth workers, in addition to best caring for physical wellbeing, must also attend to psychosocial wellbeing. Caring for Black mothers of Black boys in the U.S. demands specialized attention because Black mothers of Black boys’ experiences—manifesting in and on their bodies—demands specialized care. Caring for Black pregnant and birthing people must be simultaneously theorized and executed within an anti-racist, relationship-centered, reproductive justice framework (Hardeman et al., 2019; Julian et al., 2020). For Black midwives to meet the ever-growing demand of caring for Black pregnant and birthing people, their rightful place in midwifery and the history of the country must be restored (Goode & Katz Rothman, 2017).

The most impactful, sustainable change requires a dismantling of white supremacy and structural racism, and a complete reimagination of social systems that produce the very issues on which this paper focuses. Yet, Black midwives persist. This study’s primary limitation is the small sample size. Further studies specifically designed around this research question are needed in order to: assess the generalizability of these data and discover if there is a correlation between Black midwifery care and improved birth outcomes and experiences of Black mothers of boys. This research is essential not only because of the efficacy and affirmative nature of Black midwifery care, and the meaning-making that they bring to it, but also because their care is a form of protection and resistance to social systems that endanger Black life. The provision of unapologetic spaces for Black midwives to care for and nurture Black birthing people is healing. As Patricia (CPM, 20–25 years of experience) says, “the joy matters.”

## Data Availability

The first author maintains exclusive access to these data on a secure server, per IRB protocol.

## Footnotes

1 Copyright (c) 1996–2008, Midwifery Task Force, Inc., All Rights Reserved.

## Code Availability

The authors did not use qualitative analysis software. Coding procedures are detailed in the manuscript.
